# The Statistical Report for 1914

**Published:** 1915-11-27

**Authors:** 


### KING EDWARD'S HOSPITAL FUND.

### The Statistical Report for 1914.

(Second Notice.)

It was shown in the previous article that in the 108 hospitals dealt with in the " Blue Book," of 10,939 beds provided, only 8,963 were in 1914 in average daily use. The only metropolitan hospital of considerable size not coming in the King's Fund tables is St. Bartholomew's (757 beds), but if we were to include this unit, the percentage of unoccupied beds would show no important variation.

We are writing in times when social conditions are all at sixes and sevens, and touch hospitals in such a way that the usual gauge of economy—the average cost per occupied bed—cannot be accepted with the same degree of unquestioning confidence as in happier and more settled days. It is doubtful if the average cost of the bed occupied by a patient is ever so reliable a criterion of economy as the average cost of every person (patient or worker) fed and housed; that theory we have alluded to before and, as suggested improvements to the Statistical Report are invited, we hope to deal with it again. But, putting it at its lowest, the average cost of the bed is a guage, however rough.

The competent administrator is aware that the average cost can be lessened by careful management in the admission and discharge of patients, thereby keeping the beds constantly occupied. The continuous occupancy by a maximum number of patients naturally increases expenditure under certain headings, but the heavy standing charges remain practically at the same level, and should be lower per occupied bed.

We observe the condition of a cent. per cent. average occupancy (or very near that maximum) in about half-a-dozen instances in the pages of the Statistical Report devoted to " general particulars." Some of these are in respect of hospitals for children afflicted with incurable diseases or maladies requiring skilled treatment over a long period; in these instances the average number of days each patient was resident varied from 247 to 365 days. Even in these cases one has to stretch the imagination to understand how the annual cleaning of the wards is efficiently carried out while a maximum number of patients are retained in the building; but when we examine figures which show that the same condition prevailed in a hospital for women of 67 beds (average stay 22.6 days), and a special hospital of 70 beds (average stay 55 days), we are brought face to face with instances of hospitals where there is apparently not only skilful and ever-watchful good management in matters of prompt discharges and admissions, but considerable ingenuity in respect of periodical cleaning, decoration, and repairs. In each type of institution of which these two hospitals are representative it is a simpler matter to keep beds occupied than in those included in some other groups. The in-coming patient's name has probably been sufficiently long on a waiting list to admit of careful arrangement as to the day of admission, and to provide for negotiation with the medical authorities that there shall be a patient discharged, and a bed ready, for the newcomer's accommodation. Casualties there are none, or next to none, and emergency cases are not frequent. But there is such an extraordinary degree of precision in the idea of 100 beds always containing 100 patients for 365 days in the year, that we must plead guilty to some incredulity. Does not the figure 100 represent the establishment number of beds, the number

Average Occupancy of Beds and Stay of In-patients in Seventeen General Hospitals, 1910-1914.

| | Total Beds | 1910 | | 1911 | | 1912 | | 1913 | | 1914 | |
|---|---|---|---|---|---|---|---|---|---|---|---|
| | | Average Occupied | Average Stay | Average Occupied | Average Stay | Average Occupied | Average Stay | Average Occupied | Average Stay | Average Occupied | Average Stay |
| Charing Cross | 250 | 139 | 23 | 135 | 23 | 137 | 22 | 141 | 21 | 146 | 21 |
| German | 154 | 117 | 25 | 97* | 19 | 101* | 19 | 128 | 20 | 133 | 23 |
| Great Northern Central | 209 | 152 | 27 | 163 | 27 | 170 | 26 | 176 | 27 | 168 | 26 |
| Guy's | 644 | 533 | 22 | 545 | 21 | 539 | 21 | 526 | 20 | 522 | 20 |
| King's College | 248 | 188 | 27 | 187 | 24 | 186 | 24 | 129* | 21 | 177 | 21 |
| London | 922 | 803 | 20 | 821 | 18 | 817 | 18 | 795 | 17 | 800 | 16 |
| Metropolitan | 123 | 115 | 24 | 110 | 21 | 105 | 22 | 109 | 24 | 111 | 23 |
| Middlesex | 350 | 303 | 20 | 277 | 20 | 331 | 19 | 337 | 20 | 306 | 19 |
| Prince of Wales's | 125 | 107 | 22 | 113 | 22 | 114 | 23 | 113 | 20 | 114 | 24 |
| Royal Free | 175 | 144 | 22 | 139 | 22 | 141 | 22 | 143 | 20 | 146 | 22 |
| St. George's | 334 | No R | eturn | 294 | 23 | 318 | 23 | 317 | 23 | 314 | 24 |
| St. Mary's | 305 | 275 | 23 | 271 | 22 | 247* | 22 | 264 | 21 | 249 | 22 |
| St. Thomas's | 632 | 518 | 25 | 511 | 23 | 532 | 22 | 548 | 21 | 531 | 21 |
| Seamen's | 256 | 221 | 44 | 241 | 42 | 234 | 43 | 221 | 42 | 195 | 44 |
| University College | 305 | 274 | 25 | 277 | 24 | 279 | 24 | 280 | 24 | 266 | 22 |
| West London | 160 | 147 | 22 | 145 | 20 | 147 | 22 | 151 | 22 | 142 | 18 |
| Westminster | 213 | 184 | 25 | 170 | 25 | 181 | 25 | 183 | 23 | 174 | 25 |

* Special reason for low average.

quoted for guidance of the inquirer who wants to know the usual, officially stated, capacity of the hospital? Is not the possible number of beds—103, 104, or 105—made up of those "extras" in the middle of the ward, temporarily in the day-room, or elsewhere, with which everyone associated with hospitals is familiar? We think the solution of the mystery is probably founded on the answers to these two questions; even in that case there is little to detract from our opinion of the excellent management of a not too easy part of the work of a hospital administrator. Where there are many casualties and emergency cases, such a condition of things as that last described could not prevail. In most of the larger general hospitals, for instance, the system is one of periods of "taking in" for the different members of the staff, under which the beds of a physician are gradually emptied in readiness for his turn for receiving fresh cases during a certain number of days (when they will rapidly fill again). Under such working, naturally the unoccupied average is high, being, we believe, in the aggregate about 14 per cent. The smaller general hospitals probably work on a similar plan, and in their case, as in that of the great hospitals, a liberal margin must be allowed for a serious sudden emergency such as a large industrial mishap, or a railway accident. Most other metropolitan hospitals (excluding, in particular, for obvious reasons, the London Fever Hospital) should, with careful management and firmness in the matter of discharges, have very few unoccupied beds. Yet there are instances which are difficult to understand, such as the Cancer Hospital with only 77 beds occupied on the average out of 102; possibly the recent extensions there account for the number of vacant beds, as the new beds were not open for the whole of the year.

The table on page 185 shows at a glance the average occupation of the beds of seventeen general hospitals in five years. It also illustrates the difference in the length of stay of patients in hospitals doing the same kind of work, which varies from sixteen days at the "London" to forty-four days at the "Seamen's." We can understand the lengthy stay at the *Dreadnought* Hospitals; most of the sailor patients have no homes in London, and convalescence must be well advanced before it is safe to discharge them. In the other cases the briefness or length of stay is probably closely associated with the skill with which the almoner's department is worked, and with how much or how little the hospital is in touch with other bodies working on the principles of mutual assistance. Unfortunately, in spite of the amount that has been written and said upon the subject, there is still a great lack of co-ordination and co-operation amongst our metropolitan charities, and although there is a sufficient volume of endeavour to cause overlapping in many fields of work, much that management and skilful organisation could bring about is still left undone. It is natural that a physician or surgeon is loath to release a patient in the early stage of convalescence, to go back to surroundings where all that the hospital has effected will run a large risk of being undone, and prove so much waste of money and skilled work.

